# Association of *XPC* Gene Polymorphisms with Colorectal Cancer Risk in a Southern Chinese Population: A Case-Control Study and Meta-Analysis

**DOI:** 10.3390/genes7100073

**Published:** 2016-09-24

**Authors:** Rui-Xi Hua, Jinhong Zhu, Dan-Hua Jiang, Shao-Dan Zhang, Jiang-Bo Zhang, Wen-Qiong Xue, Xi-Zhao Li, Pei-Fen Zhang, Jing He, Wei-Hua Jia

**Affiliations:** 1 Sun Yat-sen University Cancer Center, State Key Laboratory of Oncology in South China, Department of Experimental Research, Collaborative Innovation Center for Cancer Medicine, Guangzhou 510060, Guangdong, China; mdhuarx@126.com (R.-X.H.); zhangshd@sysucc.org.cn (S.-D.Z.); zhangjb@sysucc.org.cn (J.-B.Z.); xuewq@sysucc.org.cn (W.-Q.X.); lixzh@sysucc.org.cn (X.-Z.L.); zhangpf@sysucc.org.cn (P.-F.Z.); 2 Department of Oncology, The First Affiliated Hospital of Sun Yat-sen University, Guangzhou 510080, Guangdong, China; 3 Molecular Epidemiology Laboratory and Department of Laboratory Medicine, Harbin Medical University Cancer Hospital, Harbin 150040, Heilongjiang, China; jinhongzhu625@gmail.com; 4 Department of Medical Genetics, Zhongshan School of Medicine, Sun Yat-sen University, Guangzhou 510080, Guangdong, China; jiangdh5@163.com; 5 Department of Pediatric Surgery, Guangzhou Institute of Pediatrics, Guangzhou Women and Children’s Medical Center, Guangzhou Medical University, Guangzhou 510623, Guangdong, China

**Keywords:** colorectal cancer, *XPC*, polymorphism, DNA repair, genetic susceptibility

## Abstract

Xeroderma pigmentosum group C (XPC) is a key component of the nucleotide excision repair (NER) pathway. Dysfunctional XPC protein may impair NER-mediated DNA repair capacity and further lead to genomic instability and carcinogenesis. Two common nonsynonymous polymorphisms in the *XPC* gene, Lys939Gln (rs2228001 A > C) and Ala499Val (rs2228000 C > T), have been investigated in various types of cancer. We genotyped these two polymorphisms in 1141 cases with histologically confirmed colorectal cancer (CRC) and 1173 healthy controls to explore their causative association with CRC susceptibility. Overall, no association was observed between these two variants and the risk of CRC. Our meta-analysis also confirmed a lack of overall association. Stratified analyses were performed by age, gender, smoking status, pack-year, drinking status, tumor sites, and Duke’s stages. We found that *XPC* Lys939Gln polymorphism was significantly associated with an increased CRC risk in subjects at 57 years of age or younger (adjusted odds ratio (OR) = 1.37, 95% confidence interval (CI) = 1.004–1.86, *p* = 0.047) and non-drinkers (adjusted OR = 1.53, 95% CI = 1.10–2.12, *p* = 0.011). Our results indicated that *XPC* Lys939Gln may be a low-penetrance CRC susceptibility polymorphism. Our findings warrant further validation.

## 1. Introduction

DNA damage constantly occurs because of the exposure to endogenous and exogenous mutagens. There are several DNA repair mechanisms that extensively eliminate genetic errors to maintain genome integrity, including base excision repair, mismatch repair, double-strand break repair, and nucleotide excision repair (NER). Among these DNA repair pathways, NER is in charge of repairing bulky DNA lesions, such as pyrimidine dimers, UV-induced DNA damage, chemical adducts, and crosslinks [1,2]. There are at least four critical steps involved in the NER pathway: (a) recognition of damaged DNA via a protein complex containing xeroderma pigmentosum group C (XPC); (b) uncoiling of DNA mediated by the transcription factor II human (TFIIH) complex; (c) excision of the damaged single-stranded fragment; (d) gap-filling DNA synthesis and DNA ligation [2,3].

XPC is an important protein in the NER pathway; it enables DNA repair by complexing with RAD23 homolog B (RAD23B) to facilitate the recognition of DNA damage and the initiation of DNA repair [4,5,6]. The detection of damaged DNA is the rate-limiting step in the NER pathway [5]. Therefore, the function of XPC is critical for proper DNA repair. Polymorphisms in the *XPC* gene may influence the function of the protein and an individual’s DNA repair capacity, and thereby affect genetic instability and modify individual predisposition to cancer. 

The *XPC* gene, located on chromosome 3p25, is highly polymorphic. To date, at least 102 single nucleotide polymorphisms (SNPs) have been reported in the coding region of this gene [7]. Of these coding region SNPs, *XPC* Lys939Gln (rs2228001 A > C) and Ala499Val (rs2228000 C > T) polymorphisms have been foci of interest for their potential function, as well as association with the risk of various types of cancer, including breast cancer, lung cancer, bladder cancer, and colorectal cancer (CRC) [8]. A number of studies have been conducted to explore the association between these two polymorphisms and CRC susceptibility in different ethnicities which have shown some promising results [9,10,11]. However, apart from one study with 1028 cases [12] and the current study (1141 cases), the number of cases in the included studies was relatively small, ranging from 133 to 665. These studies might be limited by low statistical power; as a result, their conclusions might not be convincing. Moreover, such an association has not been evaluated in the southern Chinese population. With this in mind, we genotyped the two SNPs and assessed their association with CRC susceptibility in 1141 CRC cases and 1173 controls recruited from southern China.

## 2. Results

### 2.1. Characteristics of Study Subjects

We analyzed in total 1141 CRC cases and 1173 healthy controls in the present study (Table 1). There was no significant difference between cases and controls (*p* = 0.518) when compared by the matching factor, gender. However, cases were significantly older than controls (55.7 ± 13.7 vs. 45.2 ± 11.6). The percentage of ever smokers in cases (27.3%) was significantly lower than that in controls (43.6%). The pack-years also differed significantly between cases and controls. Moreover, there were significantly fewer drinkers in cases than in controls. In this study, 44.3% (505) of cases had colon cancer, while 55.7% (636) had rectal cancer. In terms of the clinical stage, 130 (11.4%), 363 (31.8%), 359 (31.5%), and 289 (25.3%) patients were diagnosed with Duke’s stage A, B, C, and D colorectal carcinoma, respectively.

Genotype frequency distributions of the two SNPs in controls were in agreement with the Hardy–Weinberg equilibrium (HWE) (*p* = 0.300 for rs2228001 A > C, and *p* = 0.095 for rs2228000 C > T). Overall, neither *XPC* Lys939Gln (rs2228001 A > C) nor Ala499Val (rs2228000 C > T) polymorphism was associated with the risk of CRC (Table 2). We next combined these two polymorphisms, which might increase the effect of individual polymorphisms and improve cancer risk prediction [13]. However, we found that carriers of one or two risk *XPC* genotypes had similar cancer risk to those carrying two wild-type genotypes (adjusted odds ratio (OR) = 1.13, 95% confidence interval (CI) = 0.77–1.66, *p* = 0.524 for carriers of one risk genotype; adjusted OR = 1.03, 95% CI = 0.69–1.54, *p* = 0.884 for carriers of two risk genotypes) (Table 2).

### 2.2. Stratification Analysis

We performed a stratified analysis by age, gender, smoking status, pack-year, drinking status, tumor sites, and Duke’s stages (Table 3). A stratified analysis by age indicated that *XPC* rs2228001 A > C polymorphism was significantly associated with an increased CRC risk in subjects at 57 years of age and younger (adjusted OR = 1.37, 95% CI = 1.004–1.86, *p* = 0.047). A stratified analysis also revealed increased CRC risk associated with rs2228001 for never smokers (OR = 1.42, 95% CI = 1.05–1.92, *p* = 0.024); however, the significance of the association disappeared after adjustment for age, gender, smoking and drinking status. Moreover, non-drinkers carrying rs2228001 AA/AC genotypes showed a significantly increased risk of CRC (adjusted OR = 1.53, 95% CI = 1.10–2.12, *p* = 0.011), which was likely to be due to chance. In term of tumor sites, *XPC* rs2228001 A > C did not seem to confer predisposition to either colon cancer or rectal cancer. *XPC* rs2228001 A > C polymorphism was not associated with Duke’s stage, either. Moreover, no significant association with CRC risk was found for the rs2228000 C > T polymorphism in the stratified analysis. 

### 2.3. Meta-Analysis Results

**Figure 1:**
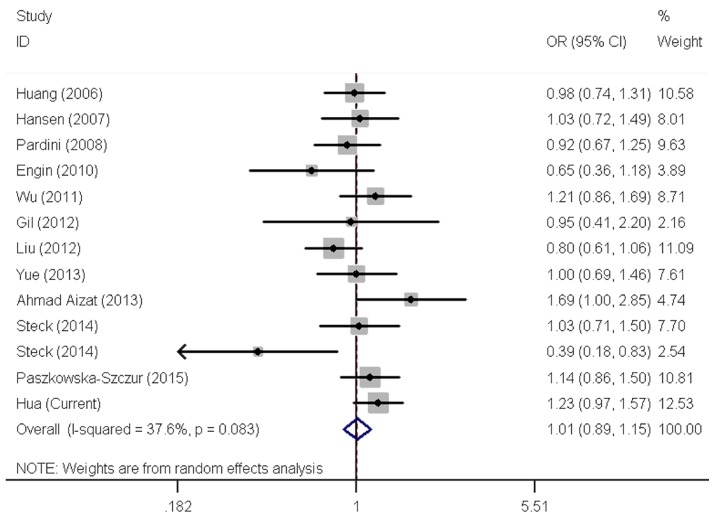
Forest plot of effect estimates for xeroderma pigmentosum group C (*XPC*) rs2228001 A > C polymorphism with overall colorectal cancer risk by a recessive model. For each study, the estimates of odds ratio (OR) and its 95% confidence interval (CI) are plotted with a box and a horizontal line. ◊, pooled ORs and its 95% CIs.

**Figure 2:**
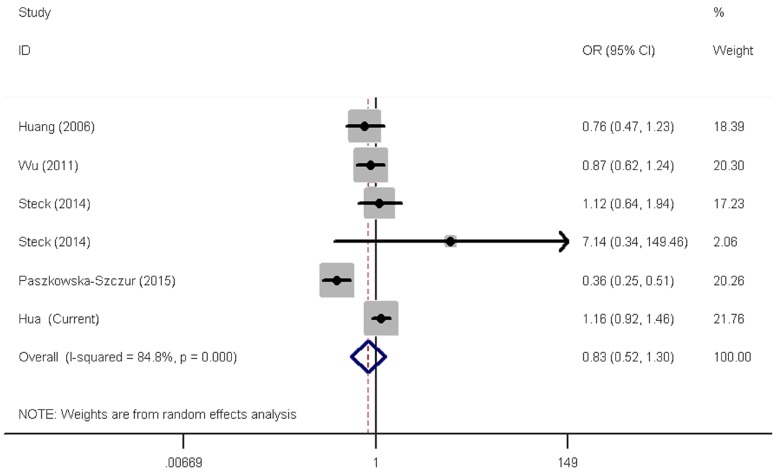
Forest plot of effect estimates for *XPC* rs2228000 C > T polymorphism with overall colorectal cancer risk by a recessive model. For each study, the estimates of OR and its 95% CI are plotted with a box and a horizontal line. ◊, pooled ORs and its 95% CIs.

The two *XPC* polymorphisms have been frequently studied for their ability to modify CRC susceptibility. However, results remain open to more than one interpretation. We next carried out meta-analysis to further explore the association between these two polymorphisms and CRC risk by including eligible publications and our data (Preferred Reporting Items for Systematic Reviews and Meta-Analyses (PRISMA) flow diagram). Overall, there were 12 publications consisting of 13 studies (6107 cases and 8207 controls) for the rs2228001 A > C polymorphism and five publications consisting of six eligible studies (3533 cases and 4860 controls) for the rs2228000 C > T polymorphism (Table 4). After pooling all studies together, risk estimates indicated a lack of correlation between the rs2228001 A>C polymorphism and CRC risk (Table 5 and Figure 1). Similarly, null association with CRC risk was found for the rs2228000C > T polymorphism in overall and stratified analyses (Table 5 and Figure 2).

## 3. Discussion

The association between two common nonsynonymous *XPC* polymorphisms (rs2228001 A > C and rs2228000 C > T) and the risk of CRC was evaluated in 1141 CRC cases and 1173 healthy controls enrolled from southern China. We found no statistical evidence of a significant association between these two polymorphisms and the risk of CRC. A combined analysis with these two polymorphisms suggested that subjects carrying one or two risk genotypes did not show increased CRC risk, when compared with non-carriers. Stratified analyses by age, gender, smoking status, pack-year, drinking status, tumor sites, and Duke’s stages demonstrated a significant association between the *XPC* rs2228001 variant allele and increased CRC risk in participants at 57 years of age or under, non-smokers, and non-drinkers. However, in the stratified analysis, age, smoking status, and drinking status had no effect on the association between the rs2228000 C > T polymorphism and the risk of CRC. The role of the *XPC* gene in carcinogenesis started to attract attention because of the observation that mutations in the DNA repair genes can cause the inherited disease xeroderma pigmentosum (XP). This disease is characterized by hypersensitivity to UV light due to impaired DNA repair capacity. XP patients have an approximately 1000-fold higher risk of developing multiple skin cancers [14]. XPC participates in NER by recognizing specific changes in the DNA structures, instead of the lesions themselves [15]. Upon recognition of damaged DNA, the XPC complex can interact with xeroderma pigmentosum group A (XPA) or TFIIH to enable the downstream steps of NER, including excision and restoration of DNA [16]. There are three widely investigated common polymorphisms in the *XPC* gene, *XPC* rs2228001 A > C, rs2228000 C > T [8], and a poly (AT) deletion/insertion on intron 9 [17]. The *XPC* rs2228001 A > C polymorphism, an A-to-C transition in exon 15, leads to a substitution of glutamine for lysine in codon 939 (Lys939Gln), while rs2228000 C > T polymorphism is a C-to-T transition in exon 8, resulting in the replacement of alanine with valine in codon 499 (Ala499Val) [18]. The *XPC* rs2228001 A > C polymorphism is located in the domain interacting with TFIIH. Both biological and biochemical evidence showed that *XPC* rs2228001 A > C polymorphism could alter the DNA repair capacity [19,20]. Moreover, lymphocytes from individuals with this *XPC* polymorphism were shown to have a reduced capacity of repairing benzo(a)pyrene DNA adducts [21].

Our findings were in line with some previous studies. A number of case-control studies have been carried out to investigate the association between *XPC* rs2228001 A > C polymorphism and the risk of CRC among Caucasians [22,23,24,25,26,27,28]. Despite biological plausibility, none of those investigations provided evidence of the association of this polymorphism with CRC susceptibility [22,23,24,25,26,27,28]. Several studies have also evaluated the role of *XPC* rs2228001 A > C polymorphism in CRC among Asians [11,12,29,30]. Wu et al. [29] reported that carriers of the CC genotype had a significantly increased risk of CRC in an eastern Chinese population with 421 CRC patients and 845 controls. Liu et al. [12] assessed the association in a northeastern Chinese population including 1028 CRC cases and 1085 controls. They found that the *XPC* rs2228001 A > C polymorphism was significantly associated with increased CRC risk under the heterozygous (OR = 1.40, 95% CI = 1.16–1.69) and dominant (OR = 1.31, 95% CI = 1.10–1.56, *p* = 0.001) genetic models. In contrast, Yue et al. [30] failed to repeat the reported association in Chinese patients in a study population of 428 CRC cases and 450 controls. The conflicting results regarding the association from different studies might be, in part, attributed to the variations in samplings, sample sizes, genotyping methods, genetic backgrounds, and ethnicities. It was also worth noting that we identified *XPC* rs2228001 A > C polymorphism as a CRC susceptibility variant in some subpopulations, including participants at 57 years of age and under, non-smokers, and non-drinkers, but these findings could lack causality. Taken together, these findings suggest that *XPC* rs2228001 A > C polymorphism may be a low-penetrance common polymorphism with a moderate effect on CRC susceptibility. Large, well-designed multicenter studies, with consideration of epidemiological characteristics and lifestyles, should be performed to clarify the role of *XPC* rs2228001 A > C polymorphism in CRC carcinogenesis.

When compared with the *XPC* rs2228001 A > C polymorphism, relatively fewer studies have examined the association of the rs2228000 C > T polymorphism with CRC susceptibility [25,27,28,29]. The results from three studies were similar to ours [25,28,29]. Interestingly, Steck et al. [28] reported a significantly positive association between this polymorphism and CRC risk in African Americans, while another study demonstrated an association in the reverse direction in Caucasians [27]. Several meta-analyses on the association between these two polymorphisms and CRC risk have been published [8,10,31]. Since then, many relevant studies have been reported. Therefore, we also performed a meta-analysis to reevaluate the association of interest, with the addition of more studies. To date, the current meta-analysis was the largest pooled study on this topic and showed null association for these two polymorphisms.

Replication study is the golden method for the validation of the genetic associations. We tested the association of interest in the study with a relatively large sample size. As a result, the statistical power was largely increased in comparison with previous studies and the results were convincing. In the present study, the statistical power to detect a significant finding in younger subjects, never smokers, and never drinkers was 77.2%, 65.4%, and 47.9%, respectively. While using our sample size, the statistical power to detect the OR of 1.20 was approximately 70% in a recessive genetic model. Therefore, this study was important in defining genetic susceptibility to CRC. Given that the genetic backgrounds of populations may vary depending on regions and ethnicities, findings from other regions and races may not be applicable to the southern Chinese population. This study was the first one to evaluate the association in the southern Chinese population. Moreover, single case-control studies often yield conflicting results, partially due to the relatively small sample sizes of each study and variations in samplings, ethnicities, genotyping methods, and study designs (e.g., population-based vs. hospital-based) among studies. To overcome these drawbacks, we also conducted a meta-analysis on the associations of interest by pooling all eligible studies and ours together. The meta-analysis results verified our findings from the current case-control study. 

However, several limitations of the studies were deserving of attention. First, the present study only had a moderate sample size. Especially, the sample size in the stratified analysis was even smaller and for this reason the statistical power might be limited. Second, we only analyzed two potentially functional polymorphisms in the *XPC* gene. More potentially functional polymorphisms in the *XPC* gene should be studied. Third, details on other risk factors, including lack of exercise, being overweight or obese, and high consumption of red meat, were not available for cases and controls, which prevented us from further investigating the effects of these parameters on the association. Fourth, the current study was a gender matched case-control study; age and other factors, such as smoking and drinking status, were not factored into the study design, which might lead to a selection bias. Fifth, an information collection bias might exist. For example, the cases had a lower percentage of individuals with a history of smoking or alcohol consumption than controls did in our current study. Sixth, 32.2% of the cases with colorectal cancer were 50 years of age or younger, which was a little higher than that (21.9%) in previous investigations performed in central China [32]. This discrepancy may be ascribed to geographical diversities. Moreover, some of our cases might have a family history of cancer. Finally, future in vitro and in vivo studies are needed to further provide biological evidence of the risk effects of *XPC* rs2228001 A > C polymorphism on the development of CRC and clarify the underlying mechanisms.

In conclusion, we did not find any predominant effects of the *XPC* rs2228001 A > C and rs2228000 C > T polymorphisms with CRC risk in the southern Chinese population. Our findings should be interpreted cautiously and warrant further validation in prospective investigations.

## 4. Materials and Methods

### 4.1. Study Population

We recruited 1141 cases with histologically confirmed CRC between January 2000 and May 2010 in Sun Yat-sen University Cancer Center. Patients with primary cancer in sites other than the colon and rectum were removed from the final study. Moreover, 1173 healthy residents in the same region were randomly recruited as controls in the same period of time, and they were frequency-matched to cases on gender. All the participants were of the ethnic Han Chinese population from southern China and genetically unrelated. Potential participants were contacted to screen for eligibility and the written informed consent was signed by each of the study subjects. A self-administered questionnaire was employed to collect information from individuals during a personal interview by their attending doctors, including demographic characteristics (age and gender), lifestyle (e.g., smoking habits and alcohol consumption), and family history of cancer. A blood sample of approximately 5 mL was obtained from each subject at the end of the interview. Overall, more than 80% of cases and controls responded. This investigation was approved by the institutional review board of Sun Yat-sen University Cancer Center (Guangzhou, China). Written informed consent was obtained from all patients.

### 4.2. Genotyping

Genotyping procedures were described elsewhere [33,34,35]. Briefly, genomic DNA was isolated from peripheral blood samples using the Qiagen Blood DNA Mini Kit (Qiagen Inc., Valencia, CA, USA). The Taqman method was chosen for genotyping these two polymorphisms with a 7900 HT sequence detector system (Applied Biosystems, Foster City, CA, USA). Quality control was strictly conducted, with four duplicate positive controls and four negative controls (omitting the DNA template) loaded in each of 384-well plates. Moreover, genotyping assays were repeated with 10% of the samples that were randomly selected and genotyping results were 100% concordant. Laboratory staff were blind to the case/control status of the samples.

### 4.3. Statistical Analysis

We performed Pearson’s *χ^2^* test and the Student’s *t* test to compare categorical variables and continuous variables between cases and controls, respectively. A goodness-of-fit *χ^2^* test was used to check the significant deviation of SNP genotype frequencies from HWE in the control subjects. An unconditional logistic regression analysis was conducted to calculate ORs and 95% CIs. An additional adjustment for age, gender, smoking and drinking status was made in the multivariate logistic regression analysis. We also carried out a stratified analysis by age, gender, pack-years, smoking and drinking status to study whether these factors could modify the association of interest. All analyses were performed using SAS software (Version 9.1; SAS Institute, Cary, NC, USA). All tests were two-sided. Results were considered statistically significant at *p* < 0.05.

### 4.4. Meta-Analysis

The meta-analyses were conducted on the association between the two SNPs (rs2228001 A > C and rs2228000 C > T) and CRC susceptibility following the PRISMA checklist [36]. Systematic literature searches of MEDLINE, EMBASE, and PubMed databases were conducted. The search terms were as follows: “*Xeroderma* *pigmentosum* group *C* or *XPC*”, “Lys939Gln or rs2228001”, “Ala499Val or rs2228000”, “polymorphism or variant or variation”, and ‘‘colorectal cancer or tumor or carcinoma or neoplasm or CRC”. Literature searches were performed between October and November 2015. Detailed procedures referred to previous publications [8,37,38,39,40]. Cross-study heterogeneity was determined by a chi-square-based *Q*-Test. The fixed-effects model (the Mantel–Haenszel method) [12] or the random-effects model (the DerSimonian and Laird method) [13] would be performed in the absence or presence of heterogeneity, respectively. Publication bias was measured by the funnel plot and the Egger’s linear regression test [14]. Furthermore, a sensitivity analysis was also performed. We performed all analyses using STATA version 11.0 (StataCorporation, College Station, TX, USA).

## Figures and Tables

**Table 1:** Clinical and demographic characteristics of colorectal cancer patients and cancer-free controls.

Variables	Cases No. (%)	Controls No. (%)	*p* ^a^
All subjects	1141 (100.0)	1173 (100.0)	
Gender			
Males	753 (66.0)	789 (67.3)	0.518
Females	388 (34.0)	384 (32.7)	
Age, year	13–89	16–80	
Mean ^b^	55.7 ± 13.7	45.2 ± 11.6	<0.0001
≤50	367 (32.2)	789 (67.3)	
51–60	342 (30.0)	285 (24.3)	
61–70	273 (23.9)	73 (6.2)	
>70	159 (13.9)	26 (2.2)	
Smoking status			
Never	830 (72.7)	662 (56.4)	<0.0001
Ever	311 (27.3)	511 (43.6)	
Drinking status			
No	968 (84.8)	600 (51.2)	<0.0001
Yes	173 (15.2)	573 (48.8)	
Pack-years			
0	830 (72.7)	662 (56.4)	<0.0001
≤30	207 (18.1)	383 (32.7)	
>30	104 (9.1)	128 (10.9)	
Sites			
Colon	505 (44.3)	-	
Rectal	636 (55.7)	-	
Duke Stages			
A	130 (11.4)	-	
B	363 (31.8)	-	
C	359 (31.5)	-	
D	289 (25.3)	-	

^a^ Two-sided chi square test for distributions between colorectal cancer cases and cancer-free controls. ^b^ Data are mean ± standard deviation.

**Table 2:** Associations between xeroderma pigmentosum group C (*XPC*) gene polymorphisms and colorectal cancer risk.

Genotypes	Cases	Controls	*p* ^a^	OR (95% CI)	*p*	Adjusted OR (95% CI)	*p* ^b^
rs2228001 A > C							
AA	476 (41.7)	472 (40.2)		1.00		1.00	
AC	497 (43.6)	557 (47.5)		0.89 (0.74–1.06)	0.172	0.92 (0.76–1.13)	0.667
CC	168 (14.7)	144 (12.3)		1.16 (0.90–1.50)	0.266	1.15 (0.86–1.54)	0.426
AC/CC	665 (58.3)	701 (59.8)	0.470 ^d^	0.94 (0.80–1.11)	0.470	0.97 (0.80–1.17)	0.752
Additive model			0.089 ^c^	1.02 (0.91–1.15)	0.734	1.06 (0.86–1.31)	0.568
AA/AC	973 (85.3)	1029 (87.7)		1.00		1.00	
CC	168 (14.7)	144 (12.3)	0.085 ^e^	1.23 (0.97–1.57)	0.086	1.20 (0.92–1.58)	0.188
rs2228000 C > T							
CC	432 (37.9)	429 (36.6)		1.00		1.00	
CT	531 (46.5)	583 (49.7)		0.91 (0.76–1.08)	0.269	0.95 (0.78–1.16)	0.621
TT	178 (15.6)	161 (13.7)		1.10 (0.85–1.41)	0.467	1.11 (0.83–1.47)	0.485
CT/TT	709 (62.1)	744 (63.4)	0.521 ^d^	0.95 (0.80–1.12)	0.521	0.99 (0.81–1.19)	0.878
Additive model			0.241 ^c^	1.01 (0.90–1.14)	0.837	1.03 (0.90–1.18)	0.688
CC/CT	963 (84.4)	1012 (86.3)		1.00		1.00	
TT	178 (15.6)	161 (13.7)	0.202 ^e^	1.16 (0.92–1.46)	0.203	1.14 (0.88–1.48)	0.327
Combined effect of *XPC* risk genotypes							
0	71 (6.2)	77 (6.6)		1.00		1.00	
1	766 (67.1)	747 (63.7)		1.11 (0.79–1.56)	0.538	1.13 (0.77–1.66)	0.524
2	304 (26.6)	349 (29.8)	0.208	0.95 (0.66–1.35)	0.756	1.03 (0.69–1.54)	0.884

CI, confidence interval; OR, odds ratio. ^a^ Chi square test for genotype distributions between cases and controls; ^b^ Adjusted for age, gender, smoking and drinking status; ^c^ for additive genetic models; ^d^ for dominant genetic models; ^e^ for recessive genetic models.

**Table 3:** Stratification analysis for associations between the *XPC* rs2228001 A > C and rs2228000 C > T genotypes with colorectal cancer risk.

Variables	rs2228001 A > C (Cases/Controls)		OR (95% CI)	*p*	Adjusted OR (95% CI)	*p* ^a^	rs2228000 C > T (Cases/Controls)		OR (95% CI)	*p*	Adjusted OR (95% CI)	*p* ^a^
	AA/AC	CC					CC/CT	TT				
Median age, years												
≤57	501/874	97/125	**1.35 (1.02–1.80)**	**0.039**	**1.37 (1.004–1.86)**	**0.047**	500/865	98/134	1.27 (0.95–1.68)	0.103	1.20 (0.89–1.62)	0.236
>57	472/155	71/19	1.23 (0.72–2.10)	0.456	1.09 (0.63–1.89)	0.749	463/147	80/27	0.94 (0.59–1.51)	0.801	0.93 (0.57–1.50)	0.755
Gender												
Males	643/689	110/100	1.18 (0.88–1.58)	0.269	1.14 (0.81–1.60)	0.445	628/685	125/104	1.31 (0.99–1.74)	0.060	1.28 (0.92–1.77)	0.140
Females	330/340	58/44	1.36 (0.89–2.07)	0.153	1.47 (0.91–2.39)	0.116	335/327	53/57	0.91 (0.61–1.36)	0.638	0.90 (0.58–1.42)	0.661
Smoking status												
Never	701/586	129/76	**1.42 (1.05–1.92)**	**0.024**	1.37 (0.96–1.95)	0.084	705/569	125/93	1.08 (0.81–1.45)	0.584	1.15 (0.82–1.61)	0.433
Ever	272/443	39/68	0.93 (0.61–1.43)	0.753	1.03 (0.65–1.64)	0.898	258/443	53/68	1.34 (0.91–1.98)	0.144	1.16 (0.75–1.81)	0.501
Pack-year												
0	701/586	129/76	**1.42 (1.05–1.92)**	**0.024**	1.37 (0.96–1.95)	0.084	705/569	125/93	1.08 (0.81–1.45)	0.584	1.15 (0.82–1.61)	0.433
≤30	175/328	32/55	1.09 (0.68–1.75)	0.720	1.00 (0.59–1.71)	0.991	170/330	37/53	1.36 (0.86–2.14)	0.194	1.13 (0.66–1.93)	0.656
>30	97/115	7/13	0.64 (0.25–1.66)	0.358	0.87 (0.32–2.38)	0.791	88/113	16/15	1.37 (0.64–2.92)	0.416	1.18 (0.53–2.62)	0.681
Drinking status												
Never	813/533	155/67	**1.52 (1.12–2.06)**	**0.008**	**1.53 (1.10–2.12)**	**0.011**	818/512	150/88	1.07 (0.80–1.42)	0.658	1.09 (0.81–1.48)	0.575
Ever	160/496	13/77	0.52 (0.28–0.97)	0.039	0.63 (0.32–1.23)	0.178	145/500	28/73	1.32 (0.82–2.12)	0.247	1.48 (0.84–2.61)	0.175
Tumor sites												
Colon	431/1209	74/144	1.23 (0.91–1.66)	0.185	1.31 (0.93–1.85)	0.124	429/1012	76/161	1.11 (0.83–1.50)	0.475	1.12 (0.80–1.57)	0.497
Rectal	542/1209	94/144	1.24 (0.94–1.64)	0.133	1.15 (0.84–1.58)	0.377	534/1012	102/161	1.20 (0.92–1.57)	0.183	1.14 (0.84–1.54)	0.394
Duke stages												
A + B	425/1209	68/144	1.14 (0.84–1.56)	0.397	1.10 (0.77–1.58)	0.593	421/1012	72/161	1.08 (0.80–1.45)	0.637	1.09 (0.77–1.54)	0.644
C + D	548/1209	100/144	1.30 (0.99–1.72)	0.059	1.31 (0.97–1.79)	0.083	542/1012	106/161	1.23 (0.94–1.61)	0.129	1.18 (0.88–1.59)	0.272

OR, odds ratio; CI, confidence interval. ^a^ Obtained in logistic regression models with adjustment for age, gender, smoking and drinking status.

**Table 4:** Characteristics of available studies included in the current meta-analysis.

Study	Year	Country	Ethnicity	Source	Genotyping	Number of Cases				Number of Controls				MAF	HWE
rs2228001 A > C						All	AA	AC	CC	All	AA	AC	CC		
Huang	2006	USA	Caucasian	PB	TaqMan	665	253	300	112	667	241	312	114	0.40	0.450
Hansen	2007	Denmark	Caucasian	PB	TaqMan	395	141	204	50	797	307	392	98	0.37	0.112
Pardini	2008	Czech	Caucasian	HB	PCR-RFLP	532	171	268	93	532	189	243	100	0.42	0.165
Engin	2010	Turkey	Caucasian	HB	PCR-RFLP	110	22	63	25	116	25	55	36	0.55	0.642
Wu	2011	China	Asian	PB	PCR-RFLP	420	155	204	61	842	363	375	104	0.35	0.639
Gil	2012	Poland	Caucasian	HB	PCR-RFLP	133	48	71	14	100	43	46	11	0.34	0.803
Liu	2012	China	Asian	PB	TaqMan	1028	360	565	103	1085	453	500	132	0.35	0.740
Yue	2013	China	Asian	HB	PCR-RFLP	428	142	225	61	450	174	212	64	0.38	0.964
Ahmad Aizat	2013	Malaysia	Asian	HB	PCR-RFLP	255	108	106	41	255	129	100	26	0.30	0.316
Steck	2014	USA	Caucasian	PB	MassARRAY	303	103	148	52	532	191	252	89	0.40	0.704
Steck	2014	USA	African	PB	MassARRAY	226	126	91	9	322	149	142	31	0.32	0.736
Paszkowska-Szczur	2015	Poland	Caucasian	PB	TaqMan	471	187	202	82	1336	480	647	209	0.40	0.711
Hua	Current	China	Asian	PB	TaqMan	1141	476	497	168	1173	472	557	144	0.36	0.300
rs2228000 C > T						All	CC	CT	TT	All	CC	CT	TT		
Huang	2006	USA	Caucasian	PB	TaqMan	689	397	261	31	703	403	259	41	0.24	0.942
Wu	2011	China	Asian	PB	PCR-RFLP	419	172	195	52	838	315	406	117	0.38	0.447
Steck	2014	USA	Caucasian	PB	MassARRAY	303	177	104	22	535	293	207	35	0.26	0.847
Steck	2014	USA	African	PB	MassARRAY	228	175	51	2	323	276	47	0	0.07	0.158
Paszkowska-Szczur	2015	Poland	Caucasian	PB	TaqMan	753	443	269	41	1288	548	563	177	0.36	0.094
Hua	Current	China	Asian	PB	TaqMan	1141	432	531	178	1173	429	583	161	0.39	0.095

HB, hospital based; PB, population based; PCR-RFLP, polymerase chain reaction-restriction fragment length polymorphism; MAF, minor allele frequency; HWE, Hardy–Weinberg equilibrium.

**Table 5:** Meta-analysis of the association between *XPC* rs2228001 A>C and rs2228001 C>T polymorphisms and colorectal cancer risk.

Variables	No. of Studies	Homozygous		Heterozygous		Recessive		Dominant		Allele Comparison	
		OR (95% CI)	*p* ^het^	OR (95% CI)	*p* ^het^	OR (95% CI)	*p* ^het^	OR (95% CI)	*p* ^het^	OR (95% CI)	*p* ^het^
rs2228001 A > C		CC vs. AA		AC vs. AA		CC vs. (AC + AA)		(AC + CC) vs. AA		C vs. A	
All	13	1.07 (0.96–1.19)	0.175	1.09 (0.96–1.24)	0.002	1.01 (0.89–1.15)	0.083	1.08 (0.97–1.21)	0.005	1.04 (0.97–1.12)	0.050
Ethnicity											
Caucasian	7	1.01 (0.87–1.18)	0.984	1.04 (0.90–1.20)	0.169	0.99 (0.87–1.14)	0.789	1.02 (0.91–1.14)	0.384	1.01 (0.94–1.08)	0.851
Asian	5	1.18 (1.02–1.38)	0.294	1.20 (0.98–1.48)	0.004	1.11 (0.89–1.39)	0.061	1.21 (1.03–1.43)	0.030	1.12 (1.03–1.21)	0.266
African	1	0.34 (0.16–0.75)	/	0.76 (0.53–1.08)	/	0.39 (0.18–0.84)	/	0.68 (0.49–0.96)	/	0.69 (0.52–0.90)	/
Source of control											
PB	8	1.04 (0.92–1.17)	0.122	1.02 (0.87–1.20)	<0.001	1.01 (0.87–1.19)	0.072	1.02 (0.88–1.18)	0.002	1.02 (0.94–1.11)	0.040
HB	5	1.16 (0.93–1.45)	0.358	1.27 (1.08–1.49)	0.995	1.00 (0.77–1.30)	0.193	1.25 (1.07–1.45)	0.924	1.11 (0.99–1.25)	0.315
rs2228000 C > T		TT vs. CC		CT vs. CC		TT vs. (CT + CC)		(CT + TT) vs. CC		T vs. C	
All	6	0.77 (0.46–1.31)	<0.001	0.91 (0.72–1.14)	<0.001	0.83 (0.52–1.30)	<0.001	0.90 (0.69–1.19)	<0.001	0.93(0.72–1.19)	<0.001
Ethnicity											
Caucasian	3	0.60 (0.26–1.36)	<0.001	0.79 (0.56–1.13)	0.001	0.66 (0.33–1.31)	0.001	0.76 (0.49–1.17)	<0.001	0.78 (0.53–1.15)	<0.001
Asian	2	0.98 (0.74–1.30)	0.195	0.90 (0.78–1.04)	0.860	1.04 (0.79–1.37)	0.182	0.92 (0.80–1.05)	0.547	0.97 (0.87–1.09)	0.261
African	1	7.88 (0.38–165.05)	/	1.71 (1.10–2.66)	/	7.14 (0.34–149.46)	/	1.78 (1.15–2.75)	/	1.75 (1.16–2.63)	/
Source of control											
PB	6	0.77 (0.46–1.31)	<0.001	0.91 (0.72–1.14)	<0.001	0.83 (0.52–1.30)	<0.001	0.90 (0.69–1.19)	<0.001	0.93(0.72–1.19)	<0.001

OR, odds ratio; CI, confidence interval; HB, hospital based; PB, population based.
